# A Report of the Method and Results of the Treatment for the Malignant Cholera, by Small and Frequently Repeated Doses of Calomel. With Numerous Illustrative Cases

**Published:** 1833-10

**Authors:** 


*A Report of the Method and Results of the Treatment for the Malignant Cholera, by small and frequently repeated Doses of Calomel; with an Inquiry into the Nature and Origin of the Complaint, with a View to the more just Appreciation of the Means for its Prevention and Cure. With numerous illustrative Cases.* By JOSEPH AYRE, M.D. London, 1833. 8vo. pp. 167.

DR. AYRE, who is favourably known as the author of two practical treatises on important subjects, has now added a third to the list, which will not detract from his well-deserved reputation. Our author, who is a resident in Hull, had a large number of cholera cases under his care, when that town was invaded by this Asiatic pestilence, last year; and the following extract from the Introduction gives a summary of his method of treatment, and its results:

"To prevent any misconception, also, as to the kind of cases admitted to be of cholera, it is to be understood that no case was recorded or reported as one, which was merely premonitory, or limited to what has been called the bilious diarrhœa, and which had not those characteristic symptoms of the disease, the gruelly or whey-like discharges, with cramps, and with the other indications equally distinctive of the disease, and of its impending or commencing collapse. In respect to the treatment, it must be borne in mind, and I therefore here repeat it, that the same mode was undeviatingly pursued with all in the stage of collapse, and that only one remedy was used for all,—that remedy being calomel, and commonly in single-grain doses, and given generally every five minutes in the severer cases, and in others at somewhat longer intervals, with a single drop of laudanum with each. No auxiliary remedies, of any kind whatever, were used in the stage of collapse, excepting a mustard cataplasm to the stomach, and bags of warm sand to the feet; and these were frequently omitted. The largest quantity of calomel taken by a patient under my care, [Case 6, an hospital patient, who recovered,] was 580 grains, and the smallest about fifteen or twenty, by an infant; the medium amount being about eighty; though many patients, and some of them children, took from two hundred to three hundred. No evil effects of any kind,—and this is a fact meriting, on many accounts, a full share of consideration, no evil effects of any kind arose, either then or since, from the medicine. Not an instance, or the semblance of an instance, of severe ptyalism occurred in any case, or where the effect continued more than a few days; and indeed it was, I believe, only to four patients (and one was a premonitory case, and not reported,) that I thought it necessary to order anything whatever to correct it.

"No patient was reported recovered who was not in a condition to walk out, and whose recovery was not entire, and did not remain so; and it will be seen that nearly all were returned cured by the fourth or fifth day, and therefore that very few were affected with consecutive fever.

"The following will be found, upon an examination of the cases, the summary of their number, and condition, and results, and of the causes which severally contributed to produce in some of them a fatal issue:

| | | |
|---|---|---|
| "Number of patients | | 219 |
| Of whom there were in extreme collapse | 86 | |
| In severe collapse | 33 | |
| In a medium degree | 46 | |
| In a mild degree, more or less advanced | 26 | |
| Having symptoms of impending collapse, with cramps and the characteristic discharges | 28 | |
| | | 219 |
| Recoveries | 176 | |
| Deaths | 43 | |
| | | 219 |
| Of the deaths | | 43 |
| There were of those who were dying when first seen, and either could not, or did not, take any medicine | 13 | |
| Of those in whom the treatment began very late, and was more or less negligently pursued | 19 | |
| In whom there was previous disease | 2 | |
| Dropsy of the brain supervening with neglect | 5 | |
| Relapse from palpable neglect in diet | 4 | |
| | | 43 |

(P. xxiii.)

Passing over the first chapter, in which the symptoms of cholera are detailed at length, we find in the second an investigation of its remote causes. These are, according to our author, a specific malaria, and poor living; the former being the exciting, and the latter the predisposing cause. Dr. Ayre is a decided anti-contagionist, and thinks that the high roads have been unjustly stigmatised as conductors of the disease, when, in fact, the rivers accompanying those high roads should bear the blame.

"What, it may be asked, upon the assumption of the disease being infectious, and of its having been brought to, and extended amongst us, by infection, is the reason why the towns of Whitby, and Scarborough, and Bridlington, and Beverley, and Hedon, with the villages lying between them, should remain free from the disease, while so many other places with which they had intercourse were affected by it. The answer plainly is, that it is not communicable; but that in the same way as the malaria producing àgue, or that occasioning the yellow fever, is generated in certain known localities, so in like manner the malaria of cholera, under atmospheric influence, is produced in certain situations, and not in others. Of the cause of this difference in the fitness of certain localities, and the unfitness of others, it is neither necessary nor easy to pronounce an opinion. It may be thought, however, not undeserving of notice, that there is one peculiarity in the towns of Goole and Gainsborough, and Selby, with York and Hull, as well as in most, if not all the other towns and villages of the country that have suffered by the disease, which distinguishes them from those of Scarborough, and Whitby, and Bridlington, and Beverley, and a multitude of others, both towns and villages, which have escaped it; and this peculiarity is, in having a navigable river running by or through them. In the track which the disease took across the continents of Asia and Europe, it was assumed to have proceeded as an infection, and by an intercommunication of persons, because it was seen to attack the inhabitants of towns placed on the high roads; but the high roads of those countries are not numerous, and they lead generally up to, and through towns which are situated on the banks of rivers. In this country, covered over as it is by its numerous population, all the roads may be said to be high roads; but the disease did not travel along them, or, if it did, it was by an arbitrary and partial choice of them; for it was not along those on which there was the most traffic, and which, according to this hypothesis, it should have done, but along those alone which terminated at towns or villages that were placed on the banks of rivers. There is a high road, and a constant intercourse between this town and Beverley, and there is the same between York and Beverley, and between this town and Scarborough and the other towns and villages of the East Riding; yet upon none of these roads did it travel to these places, or appear in them, and for the single reason that their localities were unsuited to the generation of the malaria which should produce the disease. They are distinguished from Hull, and York, and the other towns mentioned in no other discoverable circumstance than in having no rivers passing through them; but whether their exemption depended wholly, or only partially upon this cause, must be left for further and more extended observation to determine. We are at present much too uninformed of the nature of this malaria, and of the materials producing it, to decide upon what it precisely depends; or whether it is essentially, or only accidentally the produce of materials supplied by the banks of rivers. In this district it was wholly limited to places so situated; but it is quite conceivable that there may be localities apart from the banks of rivers, and yet analogous to them, which may be capable, like them, of generating the malaria of this disease. The main fact upon which it is of importance to insist, is, that the malignant cholera does not travel along roads by an intercommunication of persons, but that it owes its origin to a malaria; that this malaria, like that giving rise to ague, and to the remittents of

Walcheren and of other fenny districts, is of a specific nature, and is generated by materials supplied by rivers, or the banks of them, or by localities which are favorable to the same end, from possessing some certain properties in common with them." (P. 22.)

To this river-malaria our author adds the noxious vapours arising from drains; and mentions that, in a report drawn up by Mr. Baker, the influence of defective drainage in giving rise to the disease in Leeds is illustrated by a map, "in which it is shown that the quarters where the disease most prevailed were precisely those in which the drainage was most defective." (P. 25.) It was once supposed that insufficiency of clothing was a predisposing cause; but the disease prevailed most in the hottest weather. It was supposed, again, that the want of cleanliness in the dwellings of the poor favoured the progress of the disease; but, in the greater part of Hull, these humble tenements are not only clean, but in numerous instances remarkably neat.

"It was not therefore in the state of their clothing, nor anything in the interior arrangement or condition of their dwellings, as favouring or fostering the origin or spread of an infection, that the poor should have been the especial subjects of the disease.* There remains therefore but one other peculiarity distinguishing their condition from that of the class above them, and that is, in their diet; and in this lies the secret why the poor in this, and in all countries, endured its attacks, and why the better-conditioned classes were almost wholly exempt from it. Of all the patients whom I saw in this town, not a dozen, out of perhaps two hundred, were in circumstances to procure meat daily, and many only once or twice a-week, and some only very occasionally; while the remainder, forming an immense majority, not at all. There was indeed, among our poor, notwithstanding the appearances of cleanliness and comfort in their houses, great and varied destitution. Their food was composed of the coarsest bread and potatoes, and frequently not enough of these; both the bread and potatoes being at the same time of a bad acescent quality, and as injurious on this account as they were from the quantity required to be eaten of them to compose for the hungry a sufficient and satisfying meal. By such the stomach and system are at all times insufficiently supported, and are exposed to be affected by all the ordinary ex-

* "The author had frequent occasion, in his visits among the patients, to have it remarked to him, that the seeming comfort about their homes, as evidenced by the quality or quantity of their furniture, was in no degree real; for though, by pawning their goods, they might procure meat, yet, by being in arrears in their rent, they would be stripped by their landlords of the rest, if a single article was disposed of. They had lived and lingered on in the forlorn hope of better times for them; and it was painful to hear from the mourners, in their agony for the death of a father or husband, how the privations and self-devotion of the deceased had been the causes of their bereavement."

citing causes of disease. Such a diet, indeed, is but a mitigated species of famine; and, from its acescent and unnutritive nature, is well suited, from the disorder it induces in the digestive organs, to fit them to be acted on by the poison or malaria of the malignant cholera. And here I need scarcely remind the medical reader, that the cholera is a disease which pertains to, and commences in, the digestive organs; and that the specific malaria will act the more readily and the more powerfully upon them, if previously disturbed by an unwholesome and unnutritive diet. Hence, therefore, wherever the prevalent diet of the people consisted of an undue proportion of vegetable food, there the disease, other things being equal, prevailed the most. In Paris, from the general use of vegetable soups and dishes, and of an acescent wine, and the very defective state of its drainage, the ravages of the disease were great; and, with thousands of the poor, many of the wealthy, from the operation of these causes, became the victims of the disease. In this country, the people generally, where their means admit of it, indulge in the daily use of meat, and their soups are made of the same substantial food. A few persons only, therefore, of this class were affected; and with those who were so, it could be traced to a highly concentrated state of the malaria, or to an irregularity in their diet; as a heavy supper of some unwholesome food, or an inordinate quantity of fruit. Of all the patients whom I saw in the disease, there were but six of this class; of whom two were female servants, who from choice had abstained from meat; one had eaten for two days of indigestible ham; two had eaten inordinately of pears at supper; and one was an habitual drunkard, and had but little appetite for any food." (P. 28.)

We by no means agree with Dr. Ayre in the slur he casts on French wines: even if those consumed by the poorer Parisians are a trifle too sour, they must be far wholesomer, and far less prone to disturb the digestive organs, than narcotic porter, or cauterizing gin; and the wines consumed by all above the lowest classes are beyond all question the finest in the world—to the follower of Hygeia, as well as the disciple of Epicurus. Depend upon it, too, Dr. Ayre, that not only in this country, but in France, "the people generally, where their means admit of it, indulge in the daily use of meat, and their soups are made of the same substantial food." Indeed, all classes in tolerably easy circumstances swallow such quantities of food, including large portions of meat, that a raw stranger, sitting for the first time at a French *table d'hôte*, and gazing in speechless astonishment on the endless deglutition, is apt to become a believer in the unlimited extensibility of the human stomach, and to distrust his Soemmering, in which he reads that it will contain only from five to eleven pounds. We recollect indeed finding it stated, in the Gazette Médicale, last winter, in an ingenious article on *Choleraphobia*, that a great *gastronome*, who was also a great *choleraphobe*, caused the word CHOLERA to be placed in the inside of his snuff-box, that his progress amid the long array of dishes might be arrested by this Abernethian admonition. The number of cases, too, among the opulent classes, this summer, in London, has tended to show that the protecting power of good living is not quite so great as Dr. Ayre supposes, though it would be absurd to deny that it is very considerable; and we therefore agree with him, that a more liberal distribution of animal food among the poor would have been highly desirable.

"This was then alone the course to be followed, and for this object establishments should have been formed for the daily issue to them of rations of meat and animal soup, beginning the issue of such rations on the first appearance of the disease in a town, and continuing it during the limited period in which the epidemic influence prevailed. To lighten the burden of such measures of relief, besides the aid of public subscription, employment might have been provided for the poor in works of public improvement. Nor would the Boards of Health, in doing this, have exceeded their powers; for they were directed by the instructions issued to them by the Lords of the Privy Council, to employ every practicable means to obviate the occurrence of the disease, and to raise and apply the funds required for that purpose." (P. 38.)

In the third chapter, in which our author discusses "the Nature or Pathology of Cholera," he brings forward a number of objections to the theory which supposes cholera to arise from noxious changes in the chemical constitution of the blood, and the loss of some of its principles carried off by the discharge from the stomach and bowels. To this Dr. A. first objects, that the premonitory diarrhœa "may be arrested, and the full development of the true disease prevented, by remedies of a common kind, and which have confessedly no power to prevent or correct any change in the condition of the blood." (P. 43.) But surely this is begging the whole question: the chemical physician would say, "Two patients have the premonitory diarrhœa; one of them takes nothing, is seized with cholera, and his blood is found to have lost some of its saline constituents; the other takes rhubarb and opium, and is not seized with cholera; therefore rhubarb and opium confessedly have the power of preventing or correcting changes in the condition of the blood."

Our author proceeds to say, "The means also which act beneficially in removing the collapse, and in stopping the profuse discharges from the bowels, have no chemical properties by which to effect this change. (P. 43.) But, granting that they have no chemical properties by which to effect this change, they may produce it as vital agents; even mechanical agents are able to produce remarkable changes in animal fluids: is not the quality of the remaining blood changed by venesection, and does not a severe blow on the spine make the urine alkaline? Again, says Dr. Ayre, "In many cases they act too rapidly to admit of the supposition of their acting as chemical agents, particularly emetics, which, upon being taken, are instantly ejected from the stomach, and are thus precluded, whatever may be their composition, from effecting any changes of a chemical nature in the blood." Now, the protracted vomiting which emetics frequently cause indicates either that they are not instantly and entirely ejected from the stomach, or that their influence on the system can be kept up by habit long after every particle of the medicine has been thrown up: it is not necessary to suppose that they are absorbed into the blood; it is enough if they can modify the nervous energy, which presides not only over the circulation, but probably also over the constitution of the blood; and we can thus indirectly alter the chemical condition of the blood, just as we can correct the errors of a watch, not merely by a violent movement of the hands, but by an almost imperceptible alteration of the regulator.

Dr. Ayre proceeds to state several other objections to the chemical theory, to which it would not be very difficult to find replies; but it is needless: for the saline treatment having been tried without success, it is hardly worth while to show that the ingenious speculations on which it was founded are not so indefensible as our author imagines.

Dr. Ayre, after having demolished the saline theory, as he supposes, points out, with considerable ingenuity, the strong resemblance between the Asiatic disease and the worst forms of our old English cholera; and then gives his own theory of the nature or essence of the malignant cholera, supposing it to arise from congestion in the liver.

"Now the congestion thus produced in the portal or venous system of the liver, and in its associated organs, constitutes the stage of collapse of the cholera; and under various modifications and grades of intensity, whose real nature and amount are unknown, forms the essence of it in all. In the collapse of the disease, when rendered intense by a total and sudden cessation of the secretion of the bile, there is an abeyance of the vital powers, as the result of the venous congestion, and the disturbance which it gives to the nervous system. The pulse becomes feeble, and at length extinguished, from the twofold influence of a mechanical disturbance to the heart's action, and of the diminished energy of the brain. The surface becomes cold and of a livid hue, the eye appears sunk, and the countenance shrunken and collapsed; the secretion of the kidneys becomes diminished, and at length suppressed, from the congestion extending to the renal veins, and thus obstructing, at the same time, the action of the renal arteries; the voluntary muscles are excited into spasmodic contractions by the irritation given to the vertebral chord by the congested state of its veins; while the capillaries of the mucous surfaces of the stomach and bowels are impelled into an increased action by the stimulus of the same congestion, and profusely pour out their secretions. Now this state of the system, constituting the stage of collapse in its worst or malignant form, can only be relieved by relief being afforded to the congestion causing it. In the ordinary cases of an interruption in the secretory action of an organ, there is a provision made by nature for its relief. The congested and inflamed state of the female breast, to which a sudden cessation of its secretory functions gives rise, if not removed artificially by the local abstraction of blood, or by other means, or naturally by a renewal of its proper secretion, terminates in inflammation, and this last in suppuration, which is to the other a natural remedy, and by which the congested state of its arterial circle of vessels is relieved; while a termination is put for a time to its secretory action, which is from its nature only temporary, and a healthy state of the organ at length induced. But inflammation, and suppuration, which is one of its remedies, are the results alone of arterial action, and cannot be produced in vessels, or by the agency of vessels, whose character and structure are venous. But a permanent interruption to the secretion of the bile, and the congestion resulting from it, are conditions incompatible with life. Relief must be afforded to the collapse which the congestion creates, or death must ensue. Now the relief which is thus required may be afforded by one of two modes, but differing greatly from each other in the degree of relief which they give. The first in value and importance is a sudden reaction taking place in the secretory vessels of the liver, to which the spontaneous vomiting of the complaint not unfrequently contributes, and by which a copious secretion of bile in the milder cases of the disease is often suddenly produced. This is the usual termination of the common cholera, and the natural remedy of the complaint, although often erroneously regarded as the complaint itself; because the overflowing bile, by accidentally rising into the stomach, prolongs for a time that irritable condition of it, which the previous congested state of the liver had first induced. When this salutary reaction of the secretory vessels of the liver does not take place, and the struggle with the disease is prolonged, a second mode of relief to the collapse will take place, which is of less efficacy, and consists in a reaction of the arterial system of vessels of the structures involved in the congestion, and to which the congested state of the veins had afforded an indirect stimulus. The excited or inflamed state thus set up in these structures, and particularly in the mucous surfaces of the stomach and bowels, which, as they shared in the congestion, next partake of the increased action, forms in this, as in other cases of venous congestion, a partial but morbid remedy to it. By the inflammatory action thus established a febrile state is set up in the system, and all the symptoms of collapse disappear. The tongue, which before was moist, and perhaps cold, becomes dry and hot; the bowels no longer pour forth a copious serous secretion, but become confined, and are moved with difficulty, and their contents are laden with mucus, and of a dark, and sometimes sooty hue. The fever is termed consecutive, as following the stage of collapse, but differs in nothing from the common bilious fever of other seasons, excepting in severity, and in the circumstance of the stage of collapse in the bilious fever being occasionally too brief or too slight to be observed. In both cases the pathological conditions are the same; and mainly consist in a limited interruption, instead of an entire suppression, of the secretion of the liver, with such an increased action in the arterial circle, or system of vessels associated in the congestion, as is sufficient to force on the circulation of the blood through the congested veins, and remove, or considerably relieve, the stage of congestion and collapse. In numerous cases this relief is only partial as concerns the state of collapse; for, though the pulse may return to the wrist, and some warmth to the surface, and even to a degree sufficient to show that the circulation has become more free, this flattering state in a short time becomes succeeded by a renewed collapse, in which, if there be less coldness of the surface, there is not the less danger; for, indeed, the transition to the state of reaction in the malignant cholera is often little more than the substitution of one fearful evil for another, as many sink under it with all the signs of gastro-enteritic inflammation." (P. 58.)

In some patients, observes Dr. Ayre, a partial reaction takes place, so as to restore in a slight degree the warmth of the skin; yet they sink under a second collapse, not arising from congestion, but the result of a failure in the vital powers, and identical with the one which takes place at the fatal close of all acute diseases. In such cases the post-mortem appearances are not those of congestion, or only partially so.

This chapter is closed by several recapitulary observations, the following of which is perhaps the most important, as it is the one on which our author's practice is based: "6th. That in the cases where the congestion terminates spontaneously and *favourably*, it is by a renewal of the secretion of the bile, and frequently, in the English or common type of the complaint, by such a sudden and copious discharge of it as to occasion a copious purging and vomiting of that fluid." (P. 69.)

We now arrive at the Treatment. Dr. Ayre, after remarking that the disorder consists of three stages, viz. the premonitory diarrhœa, the stage of collapse, and the consecutive fever, dilates at some length on the first of these from p. 72 to 81, where, to our no small surprise, it makes its appearance again under the name of *the premonitory stage.* This little blemish, however, Dr. Ayre will no doubt "correct, in his second edition," as reviewers have it. The following passage gives the leading points of our author's practice in the first two stages:

"1. *The premonitory stage.* The usual dose which I gave of the calomel in this stage was one grain, united with two or three drops of laudanum, and repeated hourly, or every half-hour, for six or eight successive times, and then every six hours, or twice a-day, for a short period; directing the patients at the same time to substitute rice, in a considerable degree, for their bread and potatoes, and to take what they could procure of animal diet; enjoining them, besides, that if the disease should proceed to put on a more serious form, to begin immediately with the pills every ten or five minutes, and to acquaint me forthwith of the change. Of a very great number whom I saw in this stage of the complaint, and who were thus treated, only very few went forward in it, and required to have their cases reported.

"2. *Stage of collapse.* In the ordinary cases of the disease in this stage, I gave the calomel, as has been stated, in a single-grain dose, made into a pill with bread rubbed into a mucilage with gum-water, and so minute as to weigh, when dry, but one grain and a half, and taken every five minutes, and with it a single drop of laudanum, or Battley's sedative liquor, in a teaspoonful of cold water. In the early periods of my treating this disease, and when less experienced in it, I was led to believe that cases of it, which appeared to be mild, might be treated less actively. But I was soon undeceived in this respect; for often the mildness of it depended only upon its slower development, which at length took place, through the inefficiency of the calomel, given at wide intervals, to arrest it. The same fact was also too often forced upon my attention by the neglect of the attendants in giving the medicine regularly, by which the stage of collapse was frequently prolonged, and sometimes allowed to become most fearfully developed. In the aged this neglect was sometimes fatal, but in the young it was more easily corrected; and, on such occasions of neglect, it was a matter of agreeable surprise to observe with what effect the calomel even so given had retarded the course of the disease; and, when renewed and punctually exhibited, with what manifest power it arrested it. And here let me observe upon an objection which I have heard alleged against the practice of small and repeated doses, and which I have reason to believe has influenced many to adopt it only partially, and others wholly to reject it. The objection I refer to is, the supposed irksomeness and sickening effect to a person so ill, of taking so many pills, and that so frequently. The truth however is, that the pill, from being exceedingly minute, as it may, and should be, and placed in the cold water and swallowed with it, is readily taken; and, from the great thirst and impatient desire of the patient for cold water, is even coveted by him, and the times, as they return for having it, are even hailed by him with satisfaction. It may be also added, that with the thirst the disease abates, and with both abates also the necessity for the frequent repetition of the medicine; and thus the evil (if evil it can be called which is neither felt nor acts as one,) works out its own relief.

"In a few cases of extreme severity, I gave two grains of calomel every five minutes for an hour or two, and then resumed the ordinary dose of one grain. In giving this medicine, no other limit is required to be set to its use than that which the stage of congestion or collapse imposes; for, pending its duration, the medicine must be uninterruptedly continued, watching at the same time the decline of the disease, and widening the intervals of giving the medicine to ten, fifteen, and twenty minutes, until it becomes evident by the symptoms that this stage of the disease has passed away; for the mercurial effect of ptyalism, which is of no advantage to the complaint, will be excited if the medicine be used to any extent, either before the collapse has commenced, or after it is removed. In a very few cases only were there any ptyalism produced, and in them it was inconsiderable, and chiefly confined to the slighter kinds, and to those which were treated as premonitory, and not reported." (P. 81.)

The treatment of the consecutive fever does not offer any thing very remarkable; but Dr. A. insists strongly on the necessity of abstaining from drastic purgatives: nothing stronger than castor-oil is requisite, and that only in doses of a teaspoonful or two.

The fifth and last chapter is entirely composed of accounts of cases, and is divided into four sections. The first contains a detailed account of ten cases, all of which recovered; the second gives a detailed account of twenty-one cases, four of which died; the third section contains short notes of the remaining cases that recovered, 149 in number; the fourth section gives similar notices of the fatal cases, thirty-nine in number. The following is one of the first ten:

"CASE VI. John Vaughan, aged thirty-two, a tramp, of drunken habits. Cholera Hospital. August 14.

"Eleven P. M. Is affected with a vomiting and purging of the characteristic fluids; the skin is cold and livid; the eye sunk; the voice choleric, and the pulse extinguished at one wrist; has been affected with a diarrhœa for two days, and has only just entered the town from York; has eaten of raw grain from the fields. To have one grain of calomel with a drop of laudanum every five minutes.

15th. Ten A. M. Has been closely attended by the hospital assistant; has taken seventy pills, and a small quantity of brandy on his admission; the pulse is now quite distinguishable; the skin still cold and livid; the eye much sunk; voice very hoarse; has vomited and purged several times as before. To continue the pills.

" Two P. M. Has not been so well during the last hour; purging and vomiting continue; skin still cold and livid, but without any dampness; countenance, and voice, and pulse, still the same. To take two grains of calomel every five minutes for an hour, and afterwards one, as before. To have a broth and rice clyster, and a teaspoonful of brandy occasionally.

" Five P. M. Is better; purging stopped; sick only once. Continue the pills, omitting the laudanum.

" Nine P. M. The temperature of the skin is improved, but is still cold; the eye is also still sunk, and the voice choleric; some sickness. Pills to be continued as before, every five minutes.

" 16th. Seven A. M. Has had a good deal of sleep; the voice still choleric; pulse feeble; skin less cold and livid; purging still characteristic; has taken his pills regularly when awake; has had some beef-tea. To have a clyster of broth, and to continue the pills.

" Seven P. M. The countenance somewhat better, but the skin is still cold. Continue the pills.

" 17th. Seven A. M. Has passed a good night, and is considerably better; stools of an ash colour; has passed some urine for the first time for three days; skin of a natural heat; countenance and voice greatly improved; pulse calm. To omit the pills; to have clysters of broth and effervescing draughts.

" 18th. Has passed a good night, and declares himself to be quite well; tongue clean and moist; appetite returning.

" Eight P.M. Rejects his food from the stomach. To have eight leeches applied to his body.

" 19th. Retains his food, and is in every respect better: the stools are black.

" 20th. Is quite free from complaint, and is only detained in bed from not having clothes. Is anxious for food. This patient on the following day was able to leave the hospital, but, from having no clothes, remained a week from this time: he left us then quite well, and without any soreness of the mouth, although he took the extraordinary quantity of 580 grains of calomel between the evening of the 14th and the morning of the 17th." (P. 107.)

The following are the four fatal cases in section ii.

" CASE XXVIII. Fatal. Joseph Mason, an infant. Was attacked with water of the brain, and was in entire collapse when first seen, and got no medicine.

"CASE XXIX. Fatal. George Headley, aged thirty-four Alboro street. October 12. This patient was in a profound collapse when first seen; was visited twice during the day, and was coming out of it, when I was informed by some neighbours that he was dead, and I missed seeing him again. On the following morning early I had to pay a professional visit up the river Trent, and did not learn until the next day that he was still living. On going to him, I found him in the consecutive fever, and taking stimulants. He struggled through a fortnight, and died with symptoms of ulceration in the mucous lining of the ileum.

"CASE XXX. Fatal. Mary Allas, aged twenty-two. Alboro street. October 6. Was seized very suddenly, and the course of the disease was most rapid and violent. I saw her early after she fell into collapse. The parties who sat up with her fell asleep, and the giving the medicine was much neglected. I visited her twice in the night, and, by counting the pills, detected the omission. She died in twelve hours.

"CASE XXXI. Fatal. Eliza Crabtree, aged fifty. Scott street. October 6. Was seized in the evening, and was in extreme collapse before she was seen. At this time there had been withdrawn the privilege, previously enjoyed by me and others, of engaging, at the instant, such of the poor for hire who were willing to act as nurses to their sick neighbours. I had in this case to give one person directions to be delivered to another, who was to come to be with the sick; and in this instance, through some misdirection, instead of the seventy pills and seventy drops of laudanum left for her being given in single doses every five minutes, the whole of the laudanum was given at once, and only twelve of all the pills were taken. In the morning, when I expected to find her nearly convalescent, she was speechless and dying." (P. 125.)

It is impossible to read these, and the other fatal cases, which are narrated by Dr. Ayre with a candour which is above all praise, without being almost forced to reflect how much of the success of medical practice depends on attention to the minutest details: the intoxication of a nurse, the false information of a stupid neighbour, or the misunderstanding a verbal direction, are sufficient to nullify the results of the best-laid plan of treatment. We hope that it will be found not absolutely necessary to continue the administration of the calomel every five minutes throughout the night, as it must be impossible to obtain any large number of sleepless nurses, and without them the scheme falls to the ground.

We will now extract a few of the short cases.

"CASE XLV. Ann Naylor, aged twelve. Green Lane. Collapse of medium severity; took sixty grains; slight ptyalism; convalescent in five days. Her two brothers had died of cholera three weeks before, under the saline treatment.

"CASES XLVI. and XLVII. Ellen Watson, aged fifteen. Bella Watson, aged eight. Green Lane. Both attacked at the same time; collapse somewhat severe; each took a medium quantity of calomel; convalescent in four days.

"CASE XLVIII. Richard Watson, father to the above. Collapse, but early seen; took only about thirty grains; convalescent in three days.

"CASE XLIX. Michael Gibson, aged thirty. Newton Court, Machell Street. Collapse severe; took two grain doses of calomel every ten minutes, with laudanum; was recovered in four days; very slight ptyalism.

"CASE L. Mary Bransby, aged twenty. Green Lane. Collapse severe; urine suppressed seventy-six hours; took about 150 grains of calomel; recovered in five days.

"CASE LI. Mary Hall, aged thirty. Green Lane. A sweep's wife. Collapse of medium severity; took about sixty grains of calomel." (P. 129.)

Dr. Ayre is so persuaded of the efficacy of his plan, he is so zealous a calomelarian, that he thinks it necessary to offer a sort of apology for each of the unsuccessful cases; as, for example:

"CASE CXCVII. E. Smith. Wincolmlee. This was a young married woman, who, after recovering from a most severe attack of the disease, relapsed into it from eating too hearty a supper at the house of her father, and sank, at the end of a fortnight, from the exhaustion caused by a miscarriage, which the second attack of the disease had produced. No second case.

"CASE CXCVIII. Plumstead, aged forty. A watchman. Mill Street. I saw this case at Mr. Sharpe's request. He was brought out of the collapse, and appeared, during several days, to be recovering; when, unknown to us, he had ginger beer and other improper things given to him, and he died from an inflammation of the mucous surfaces. No second case.

"CASE CXCIX. Jane Henderson, aged twenty-two. Bellamy's Square. This was a young married woman, whose husband was at sea, and who, after exerting herself with the most distinguished humanity and zeal in her gratuitous personal aid to the very numerous sick of the square she lived in, became herself the subject of the disease, and at length, the victim of it. I saw her in the collapse, but early in it, and she had become convalescent; when, being hungry, and having no other food, she ate for her supper the half of a flour-and-water dumpling; relapsed early in the night; and in the morning, when I first learnt of her relapse and saw her, she was just expiring. She merited a better fate." (P. 151.)

We confess that half of a flour-and-water dumpling seems scarcely an adequate cause for a relapse, and ginger beer no cause at all: call it *Haustus effervescens cum Pulv. Zingib.*

*gr. iij.*, and it might not only be tolerated, but prescribed. "The other improper things," however, cannot be defended.

We have now given a very copious analysis of Dr. Ayre's work, with but little commentary of our own; for such a book does not require it. The facts and figures speak very forcibly, whatever may be thought of the theory with which they are connected; and a mortality of only one in five, in so large a number of cases, must procure our author a host of proselytes. We certainly think that the two most plausible methods of treating cholera hitherto proposed, have been to stimulate the liver with calomel, or to administer the sulphate of quinine, considering the disease simply as an intense ague.

